# Effects of a Self-Developed Composite Modifier on the Compressive Strength, Shrinkage Behavior, and Microstructure of Cement-Stabilized Macadam

**DOI:** 10.3390/polym18202470

**Published:** 2026-10-10

**Authors:** Dawei Ma, Pengyi Wan, Yong Huang, Wenrui Yang, Zhaoguo Liu, Qi Li

**Affiliations:** 1 Nanchang Construction Investment Group Co., Ltd., Nanchang 330038, China; 2 Jiangxi Port and Shipping Construction Investment Group Co., Ltd., Nanchang 330000, China; 3 School of Civil and Architectural Engineering, East China University of Technology, Nanchang 330013, China

**Keywords:** cement-stabilized macadam, drying shrinkage, DX-3 composite modifier, microstructure, temperature shrinkage

## Abstract

Cement-stabilized macadam is widely used in pavement base layers but is susceptible to shrinkage-induced cracking. This study evaluated a self-developed composite modifier, designated DX-3, with the aim of improving volumetric stability while maintaining compressive strength. Mixtures with cement dosages of 4.0%, 4.5%, and 5.0% and DX-3 dosages of 0–2.0% were tested for unconfined compressive strength (UCS), drying shrinkage, temperature shrinkage, and microstructural characteristics using SEM and EDS. The UCS generally increased with DX-3 dosage up to 1.5% and then stabilized or slightly decreased. At 4.5% cement and 1.5% DX-3, the 7 d and 28 d UCS reached 5.67 and 7.43 MPa, respectively. Adding 1.5% DX-3 reduced the total drying shrinkage coefficient by 21.54–30.93%, the average temperature shrinkage coefficient by 2.71–3.72%, and the peak coefficient by 3.45–4.58%. The greatest temperature shrinkage sensitivity occurred at 0 to −10 °C. SEM observations indicated a more continuous cementitious matrix with more gel-like microstructural features after DX-3 modification. Overall, DX-3 improved shrinkage-related volumetric stability without compromising basic compressive strength.

## 1. Introduction

Cement-stabilized macadam is widely used as a pavement base material because of its high strength, high stiffness, good integrity, and relatively low construction cost [1,2,3,4,5,6]. However, its quasi-brittle nature and pronounced volumetric shrinkage remain major concerns for long-term pavement performance. During cement hydration, moisture loss, and temperature variation, restrained shrinkage can generate tensile stresses within the base layer. Once these stresses exceed the tensile capacity of the material, microcracks may initiate and gradually propagate. Under repeated environmental and traffic loading, such cracks can further extend upward into the asphalt surface layer and eventually develop into reflective cracking, thereby accelerating pavement deterioration and shortening service life [7,8,9,10]. Therefore, improving the cracking resistance of cement-stabilized macadam requires not only sufficient mechanical strength but also better shrinkage resistance and deformation compatibility.

Material modification has been widely investigated as an effective approach to improve the cracking resistance of cement-stabilized macadam. Existing modifiers can generally be classified into liquid or polymer-based additives and solid admixtures. Water-reducing agents, polymer emulsions, and other chemical additives can modify particle interaction and cementitious bonding, while rubber particles, fibers, expansive agents, and steel slag have been introduced to improve deformation capacity or volume stability [11,12,13,14,15,16,17,18,19,20,21,22,23,24,25]. For example, appropriate water-reducing agents have been reported to reduce shrinkage-related cracking, whereas polymer modifiers can improve the bonding characteristics and mechanical performance of cement-stabilized mixtures [17,18,19]. Rubber particles and fibers can enhance deformation capacity and delay crack development, although excessive incorporation may adversely affect stiffness or strength [20,21,26,27,28]. Other mineral or composite admixtures, such as steel slag and early-strength anti-cracking materials, have also shown potential for improving mechanical properties and shrinkage resistance [29,30].

Although polymer emulsions, rubber particles, fibers, mineral admixtures, and expansive or anti-shrinkage agents have been investigated for cement-stabilized macadam, several issues remain unresolved. First, many existing modification strategies primarily target a single property, such as strength, toughness, crack resistance, or shrinkage compensation, and improvements in deformation capacity may be accompanied by losses in stiffness or load-bearing capacity. Second, drying shrinkage and temperature shrinkage are frequently considered separately, although pavement bases are simultaneously subjected to moisture and temperature variations in service. Third, the connection between macroscopic strength–shrinkage responses and the associated evolution of the cementitious matrix remains insufficiently understood. Therefore, there remains a research need to develop and evaluate a modification approach that can simultaneously maintain mechanical strength, reduce both moisture- and temperature-induced shrinkage sensitivity, and establish a microstructural basis for the observed macroscopic behavior.

The primary scientific innovation of this study lies in developing a composite modification strategy aimed at improving shrinkage resistance without sacrificing the load-bearing capacity of cement-stabilized macadam. Unlike conventional approaches that primarily rely on a single flexible, reinforcing, or shrinkage-compensating component, DX-3 combines a polyether–carboxylic polymer system with cellulose, polymer nanomaterials, and auxiliary components to regulate the cementitious matrix through complementary effects. Therefore, DX-3 is expected to reduce moisture- and temperature-induced shrinkage by mitigating local deformation concentration and improving volumetric stability without significantly compromising mechanical strength. However, the effects of DX-3 dosage on the mechanical and shrinkage behavior of cement-stabilized macadam, as well as the associated microstructural changes, have not yet been systematically investigated.

Accordingly, this study systematically investigates the effects of cement dosage and DX-3 dosage on the performance of cement-stabilized macadam. Unconfined compressive strength tests were first conducted at different curing ages to evaluate whether the incorporation of DX-3 adversely affects the load-bearing capacity of the material and to identify a suitable dosage range. Drying shrinkage and temperature shrinkage tests were subsequently performed to assess its influence on volumetric stability and shrinkage-related performance. In addition, scanning electron microscopy coupled with energy-dispersive X-ray spectroscopy (SEM-EDS) was employed to characterize changes in hydration-product morphology, interfacial features, and elemental composition. By combining macroscopic performance tests with microscopic observations, this study investigates whether DX-3 can reduce drying- and temperature-induced shrinkage without compromising the compressive strength of cement-stabilized macadam and tests the hypothesis that an appropriate DX-3 dosage can reduce shrinkage sensitivity while maintaining adequate mechanical strength, accompanied by a more continuous cementitious matrix.

**Figure 1:**
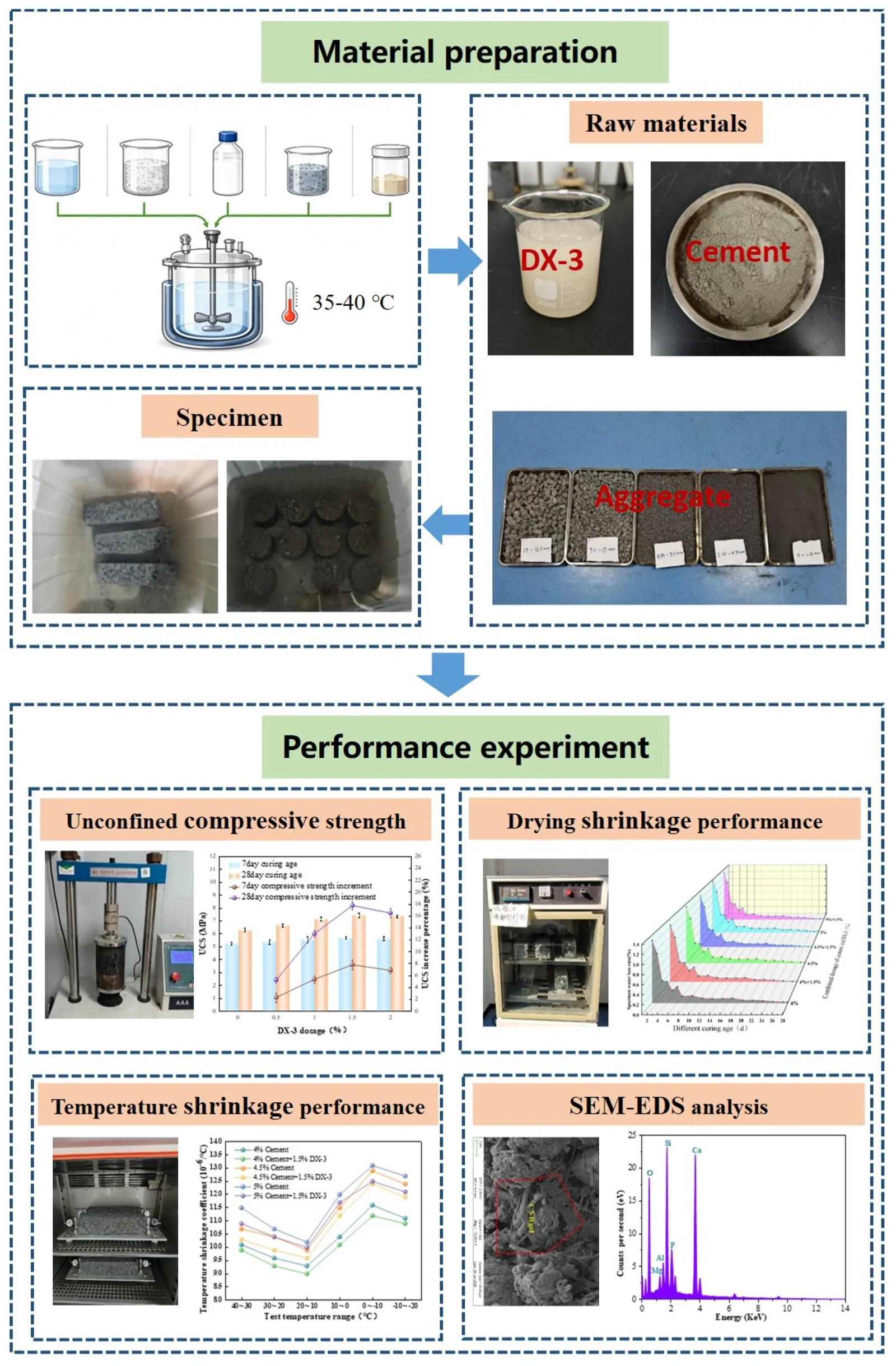
Technical flowchart of the study.

The technical flowchart is shown in Figure 1.

## 2. Materials and Methods

### 2.1. Materials

#### 2.1.1. Cement

Ordinary Portland cement (P.O. 42.5) supplied by Jiangxi Shanggao Southern Cement Co., Ltd. (Yichun, China), was used in this study. Its physical properties are shown in Table 1.

#### 2.1.2. Aggregate

The aggregates used in this test included coarse aggregates—material 1# (19–31.5 mm), material 2# (9.5–19 mm), and material 3# (4.75–9.5 mm)—and fine aggregates—material 4# (2.36–4.75 mm) and material 5# (0–2.36 mm). All aggregates were supplied by Jiangxi Basalt Mining Co., Ltd. (Ganzhou, China). The properties of the crushed aggregates are listed in Table 2.

#### 2.1.3. DX-3 Composite Modifier

DX-3 is a self-developed composite modifier, and all raw materials used for its preparation were commercially available industrial-grade materials. The main constituents of DX-3 include methallyl polyoxyethylene ether, methacrylic acid, cellulose, polymer nanomaterials, propylene glycol, and other composite additives. The development of DX-3 was based on a multi-component synergistic modification concept. The underlying design principle was to avoid relying on a single mechanism for shrinkage mitigation and instead combine polymeric and auxiliary components with complementary functions to regulate the development and continuity of the cementitious matrix. A polyether–carboxylic polymer was used as the primary polymeric component, while cellulose, polymer nanomaterials, propylene glycol, and other auxiliary constituents were incorporated to form a composite modification system. Prior to the experimental investigation, it was hypothesized that an appropriate dosage of DX-3 could improve the continuity and deformation compatibility of the cementitious matrix and consequently reduce its sensitivity to moisture- and temperature-induced shrinkage, while maintaining the compressive strength required for pavement base applications.

Methallyl polyoxyethylene ether was supplied by Shandong Qiangsen Chemical Co., Ltd. (Zibo, China); methacrylic acid was obtained from Nantong Chenrun Chemical Co., Ltd. (Nantong, China); the polymer nanomaterial was obtained from Shandong Yukang Chemical Co., Ltd. (Jinan, China); propylene glycol was supplied by Tangshan Sanyou Group Xingda Chemical Fiber Co., Ltd. (Tangshan, China); and ammonium persulfate (APS) was obtained from Guangzhou Yongliwang Trading Co., Ltd. (Guangzhou, China). 

During preparation, deionized water was first added to a reaction vessel, and methallyl polyoxyethylene ether was introduced at 40–45 °C under stirring at approximately 400 rpm for 20 min until it was completely dissolved. The temperature was then raised to 60 ± 2 °C, and a methacrylic acid solution, together with an ammonium persulfate (APS) initiator solution, was slowly added dropwise under continuous stirring over approximately 1 h. After the addition was completed, the reaction was maintained for another 1.5 h to obtain a homogeneous polyether–carboxylic polymer mother liquor.

**Figure 2:**
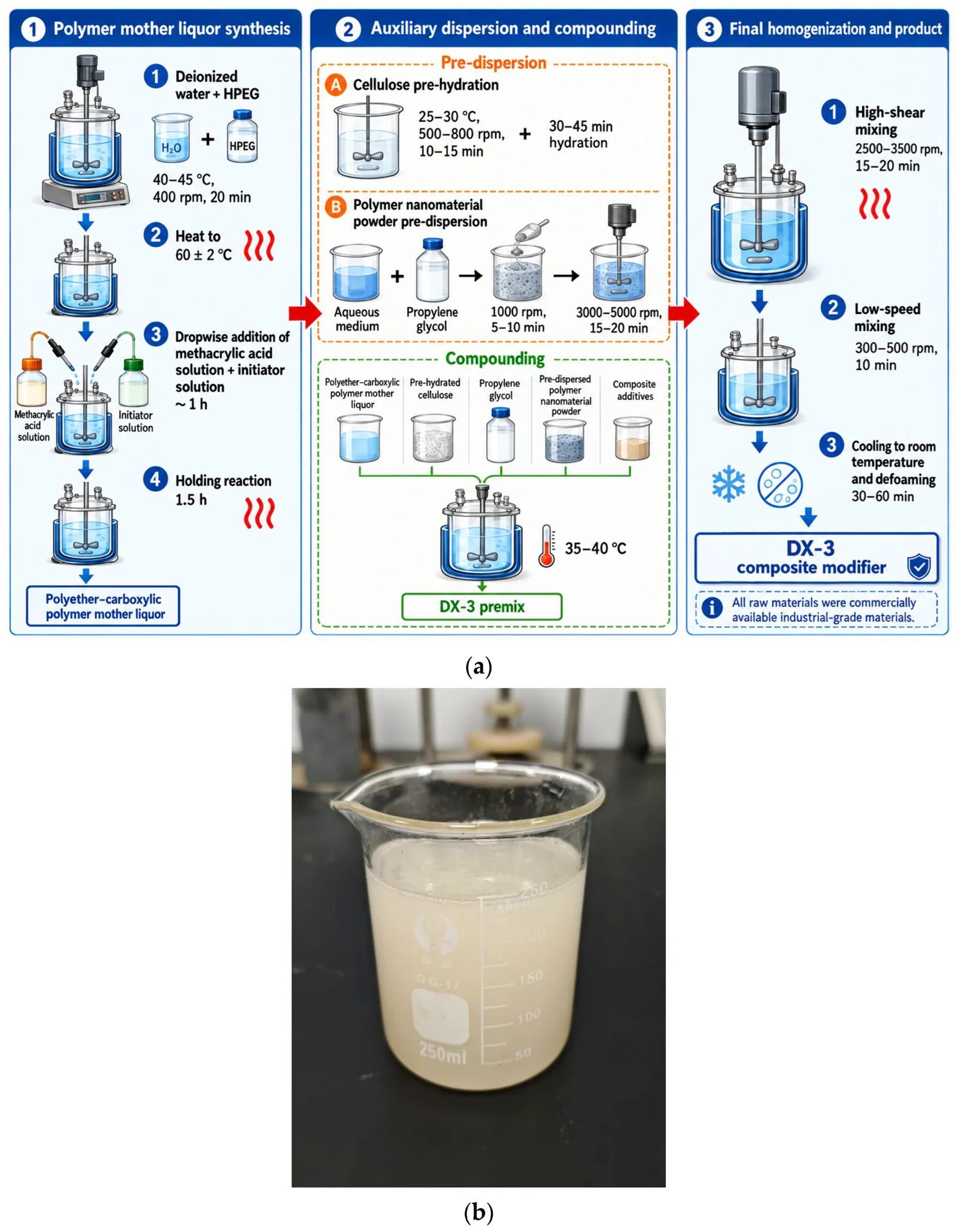
Preparation and appearance of the DX-3 composite modifier: (**a**) schematic illustration of the preparation process; (**b**) photograph of the DX-3 composite modifier (Numbers indicate the sequential preparation steps, and arrows indicate the process flow; A and B denote cellulose pre-hydration and polymer-nanomaterial pre-dispersion, respectively).

After the polymerization reaction, the system was cooled to 35–40 °C. The cellulose was pre-hydrated and dispersed before being slowly added to the polymer mother liquor, followed by the addition of propylene glycol for compounding. The polymer nanomaterial powder was pre-dispersed in an aqueous medium and then incorporated into the mother liquor under high-shear mixing. After all components had been added, the mixture was sheared at approximately 2500–3500 rpm for 15–20 min, followed by further stirring at a reduced speed for about 10 min to ensure sufficient mixing and uniform dispersion. Finally, the system was cooled to room temperature and allowed to stand for defoaming, thus yielding the DX-3 composite modifier. The preparation process and an actual photograph of DX-3 are shown in Figure 2. The schematic illustration of the DX-3 preparation process shown in Figure 2 was generated with the assistance of ChatGPT Images 2.0. The tool was used solely for visualizing the experimental preparation procedure described in this section and was not used to generate experimental data, modify experimental results, or provide scientific interpretation. The prompt used for image generation was: “Create a scientific schematic illustration showing the preparation process of a composite polymer modifier (DX-3), including polymerization, component mixing, high-shear dispersion, cooling, and the final modifier product. The illustration should be suitable for a scientific manuscript and clearly represent the experimental workflow.” The generated image was subsequently reviewed, edited, and verified by the authors to ensure consistency with the actual laboratory procedure. The approximate composition ranges of the main constituents are summarized in Table 3. The physicochemical properties of the prepared DX-3 are listed in Table 4.

### 2.2. Mix Design and Specimen Preparation

#### 2.2.1. Target Gradation

**Figure 3:**
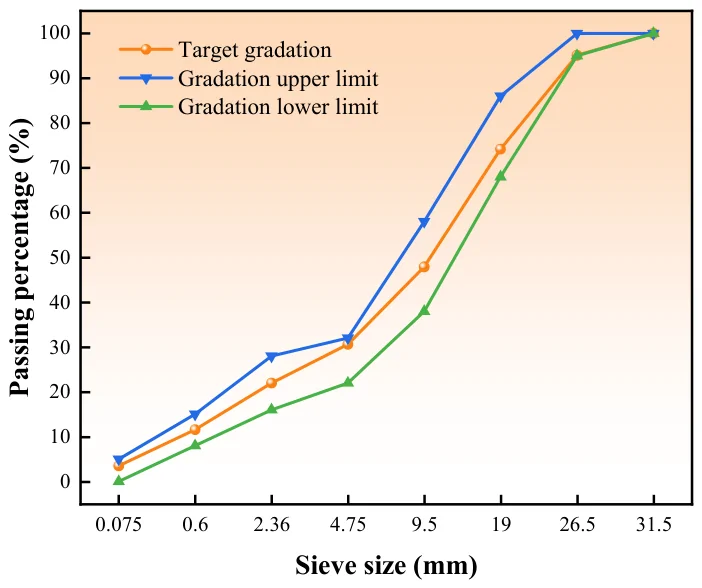
Designed aggregate gradation and specification limits.

The aggregate gradation was designed according to the requirements for cement-stabilized macadam specified in the Technical Guidelines for Construction of Highway Roadbases (JTG/T F20—2015). Based on the screening results of the five aggregate fractions, the mass proportions of 1#, 2#, 3#, 4#, and 5# aggregates were determined as 28%, 27%, 17%, 6%, and 22%, respectively. The resulting aggregate gradation satisfied the specified gradation range, as shown in Figure 3. This gradation was used consistently for all subsequent mixture preparation and performance tests.

#### 2.2.2. Compaction Characteristics

To determine the optimum moisture content and maximum dry density of cement-stabilized macadam with different cement dosages and to provide a basis for subsequent specimen preparation, heavy compaction tests were conducted using a JZ-2D heavy compaction apparatus (Shanghai Rongjida Instrument Technology Co., Ltd., Shanghai, China) in accordance with JTG 3441-2024 [31].

Before testing, the five aggregate fractions were weighed according to the designed gradation and thoroughly mixed. For cement dosages of 4.0%, 4.5%, and 5.0%, five moisture contents were selected for each mixture, with an interval of 0.5% between adjacent levels. The aggregates were thoroughly mixed at the predetermined moisture content to ensure a uniform moisture distribution. The corresponding amount of cement was then added and rapidly mixed until homogeneous, followed by compaction according to the specified procedure. After compaction, the wet density and actual moisture content of each specimen were determined, and the corresponding dry density was calculated. The test results are presented in Table 5.

To minimize the influence of compaction variability on the subsequent mechanical and shrinkage tests, all specimens with the same cement content were prepared using the same compaction-control criterion. A target compaction degree of 98% was adopted based on the corresponding maximum dry density obtained from the compaction test. Accordingly, the target dry densities were 2.394, 2.402, and 2.432 g/cm^3^ for cement contents of 4.0%, 4.5%, and 5.0%, respectively. The same compaction criterion was applied to all DX-3 dosage levels within each cement-content group. The achieved dry densities and corresponding degrees of compaction were also determined for each mixture. As summarized in Table 5, the compaction degrees ranged from approximately 97.9% to 98.2%, indicating that mixtures with different DX-3 dosages attained comparable compaction states.

As shown in Table 6, a one-way analysis of variance (ANOVA) was used to examine the effect of DX-3 dosage on specimen dry density. No statistically significant difference in dry density was observed among the investigated DX-3 dosage levels (F(4,10) = 0.0075, *p* = 0.9999). This result suggests that, under the adopted compaction-control procedure, variation in DX-3 dosage did not produce a measurable change in specimen density.

#### 2.2.3. Specimen Preparation

Based on the optimum moisture content and maximum dry density determined from the compaction tests, the required masses of aggregate, cement, water, and DX-3 were calculated for each mixture. The five aggregate fractions were oven-dried, weighed according to the designed gradation, and thoroughly mixed. Cement was then added at dosages of 4.0%, 4.5%, and 5.0% by mass of aggregate, while DX-3 was incorporated at dosages of 0%, 0.5%, 1.0%, 1.5%, and 2.0% by mass of cement. These cement contents were selected because they represent commonly adopted ranges for cement-stabilized macadam base materials. The selected range also allows evaluation of interactions among cement dosage, mechanical performance, and shrinkage behavior. The water content of each mixture was adjusted to the corresponding optimum moisture content, after which the materials were mixed thoroughly and molded immediately. The water contained in the liquid DX-3 modifier was considered as part of the total mixing water. The amount of externally added water was adjusted according to the optimum moisture content determined from the compaction test. The mixing sequence was kept identical for all mixtures to minimize variability associated with specimen preparation. The complete mixture proportions are summarized in Table 7.

**Figure 4:**
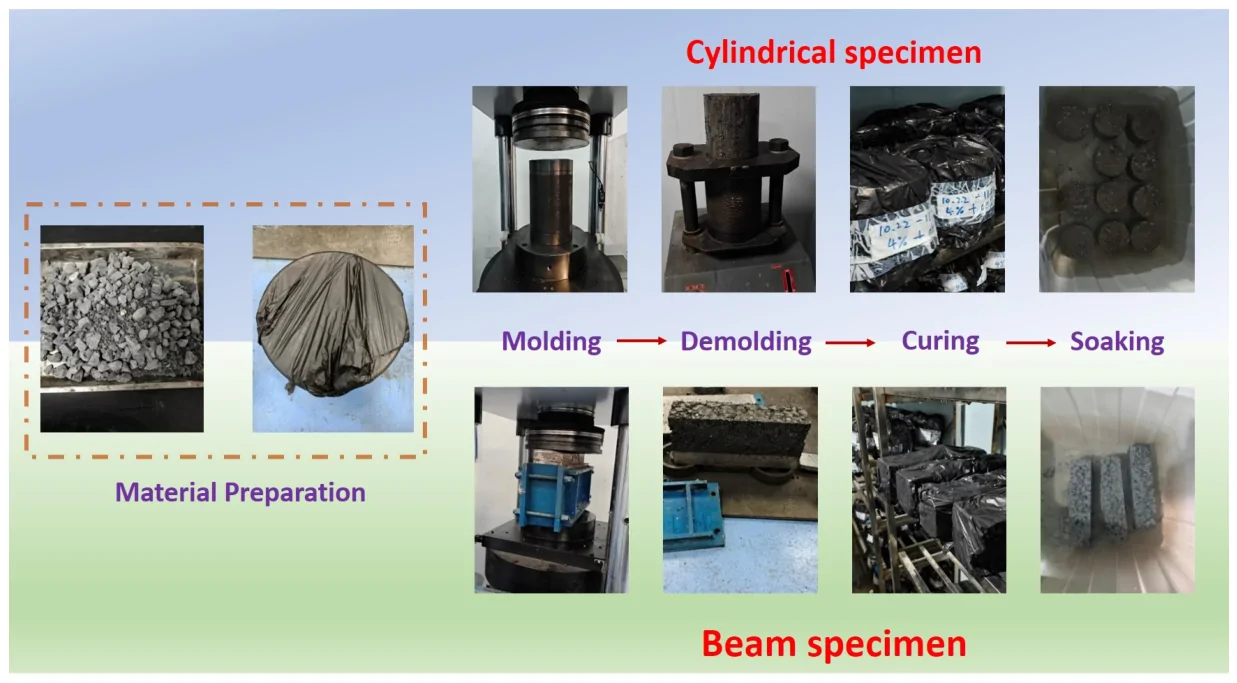
The specimen preparation process.

Cylindrical specimens with dimensions of Φ150 mm × 150 mm were prepared for the unconfined compressive strength test, whereas prismatic specimens with dimensions of 100 mm × 100 mm × 400 mm were prepared for the drying shrinkage and temperature shrinkage tests. After molding, the specimens were demolded after 4 h and placed in plastic bags. Subsequently, the specimens were cured under standard conditions at 20 ± 2 °C and relative humidity ≥95%. During the final 24 h of the curing period, the specimens were immersed in water at 20 ± 2 °C before testing. The specimen preparation process is shown in Figure 4.

### 2.3. Methods

To comprehensively evaluate the influence of DX-3 on cement-stabilized macadam, the experimental program focused on three aspects: mechanical strength, shrinkage-related volume stability, and microstructural characteristics. The UCS test was used to evaluate the basic load-bearing capacity; drying and temperature shrinkage tests were employed to characterize moisture- and temperature-induced volumetric deformation, respectively; and SEM-EDS analysis was conducted to provide microscopic evidence for the observed macroscopic performance changes.

#### 2.3.1. Unconfined Compressive Strength Test

The unconfined compressive strength (UCS) test was conducted to evaluate the load-bearing capacity of cement-stabilized macadam and to determine whether the incorporation of DX-3 adversely affected its basic mechanical strength. The experimental equipment was from Hebei Jianyi Instrument Equipment Co., Ltd. (Cangzhou, China)**.** Cylindrical specimens with dimensions of Φ150 mm × 150 mm were tested after curing for 7 d and 28 d. Three cement dosages of 4.0%, 4.5%, and 5.0% and five DX-3 dosages of 0%, 0.5%, 1.0%, 1.5%, and 2.0% by mass of cement were considered. For each combination of cement dosage, DX-3 dosage, and curing age, 13 replicate specimens were prepared and tested. Therefore, 13 specimens were evaluated for each mixture at 7 d and another 13 specimens at 28 d. Before UCS testing, the specimens were conditioned according to Test Methods of Materials Stabilized with Inorganic Binders for Highway Engineering (JTG 3441-2024). After curing to the specified age, the specimens were immersed in water at 20 ± 2 °C for the final 24 h of the curing period. After removal from water, excess surface moisture was wiped off, and the specimens were immediately tested under unconfined compression until failure. The loading rate was set to 1 mm/min to ensure a stable loading process and comparable test results. The UCS was determined from the maximum failure load and the cross-sectional area of the specimen; the calculation formula is shown in Equation (1).

(1) $$R_{c}=\frac{P}{A}=\frac{4P}{\pi D^{2}}$$

where *R_c_* is the unconfined compressive strength (MPa); *P* is the maximum load at failure (N); *A* is the cross-sectional area of the specimen (mm^2^); and *D* is the specimen diameter (mm).

#### 2.3.2. Drying Shrinkage Test

The drying shrinkage test was conducted to evaluate the volumetric stability of cement-stabilized macadam under moisture-loss conditions and to assess the effect of DX-3 on drying-shrinkage-induced cracking potential. Based on the UCS screening results, 1.5% DX-3 was selected as a representative dosage that maintained relatively high compressive strength while limiting unnecessary modifier addition. Specimens with cement dosages of 4.0%, 4.5%, and 5.0%, with and without 1.5% DX-3, were tested for comparison. Six replicate specimens were prepared for each combination, maturation period, and performance metric, with three specimens used for shrinkage deformation measurement and three specimens used for moisture loss measurement.

After curing, the specimens were transferred to a constant-temperature and humidity chamber (LHS-150HC-II, Shanghai Yiheng Scientific Instrument Co., Ltd., Shanghai, China) maintained at 20 ± 0.5 °C and a relative humidity of 60 ± 5%. The initial mass and length of each specimen were recorded before drying. During the drying process, changes in specimen mass and longitudinal deformation were measured at specified intervals. The water-loss rate, drying shrinkage strain, and drying shrinkage coefficient were subsequently calculated to evaluate the effect of DX-3 on the drying shrinkage behavior of cement-stabilized macadam. The calculation formulas are shown in Equations (2)–(5).

(2) $$\omega_{i}=\frac{m_{i}-m_{i+1}}{m_{p}}\times 100$$

(3) $$\varepsilon_{\mathrm{di}}=\frac{\delta_{\mathrm{di}}}{l}\times 10^{6}$$

(4) $$\alpha_{\mathrm{di}}=\frac{\varepsilon_{\mathrm{di}}}{\omega_{i}}$$

(5) $$\alpha_{d}=\frac{\sum_{i=1}^{n}\varepsilon_{\mathrm{di}}}{\sum_{i=1}^{n}\omega_{i}}$$

where *ω_i_* is the water-loss rate during the *i*-th measurement interval (%); *m_i_* and *m_i_*_+1_ are the specimen masses at two consecutive measurements (g); *m_p_* is the oven-dry mass of the specimen after completion of the test (g); *δ_di_* is the drying shrinkage displacement during the *i*-th measurement interval (mm); *l* is the initial specimen length (mm); *ε_di_* is the drying shrinkage strain during the *i*-th interval (με); *α_di_* is the drying shrinkage coefficient during the *i*-th interval (με/%); and *α_d_* is the total drying shrinkage coefficient (με/%).

#### 2.3.3. Temperature Shrinkage Test

The temperature shrinkage test was performed to characterize the deformation sensitivity of cement-stabilized macadam to temperature variations and to evaluate the effect of DX-3 on thermal-shrinkage-induced cracking potential. Three replicate specimens were prepared for each combination, maturation period, and performance metric. Specimens with cement dosages of 4.0%, 4.5%, and 5.0%, with and without 1.5% DX-3, were selected for comparison.

The specimens were placed in a programmable high–low temperature chamber (SZ-80, Shuyang County Municipal Instrument Co., Ltd., Suqian, China), and the temperature was varied stepwise from 40 °C to −20 °C at intervals of 10 °C. In accordance with JTG 3441—2024 (T0855—2024), the temperature was decreased at a rate of 0.5 °C/min. After each target temperature was reached, the specimens were maintained under constant-temperature conditions for 3 h. The longitudinal deformation of each specimen was recorded at the end of each constant-temperature stage, and the temperature shrinkage coefficient was calculated for the corresponding temperature interval to characterize the sensitivity of the mixtures to temperature-induced deformation. The calculation formulas are shown in Equations (6)–(9).

(6) $$\varepsilon_{\mathrm{ti}}=\frac{\delta_{\mathrm{ti}}}{l}\times 10^{6}$$

(7) $$\alpha_{\mathrm{ti}}=\frac{\varepsilon_{\mathrm{ti}}}{\left|T_{i}-T_{i+1}\right|}$$

(8) $${\bar{\alpha }}_{t}=\frac{1}{n}\sum_{i=1}^{n}\alpha_{\mathrm{ti}}$$

*α_t_*_,*peak*_ = max(*α_t_*_1_, *α_t_*_2_, …, *α_tn_*) (9)

where *δ_ti_* is the temperature shrinkage displacement during the *i*-th temperature interval (mm); *l* is the initial specimen length (mm); *ε_ti_* is the corresponding temperature shrinkage strain (με); *T_i_* and *T_i_*_+1_ are two consecutive temperature levels (°C); *α_ti_* is the temperature shrinkage coefficient for the *i*-th temperature interval (με/°C); *n* is the number of temperature intervals; ${\bar{\alpha }}_{t}$ is the average temperature shrinkage coefficient (με/°C); and *α_t_*_,*peak*_ is the maximum temperature shrinkage coefficient among all investigated temperature intervals (με/°C).

#### 2.3.4. SEM-EDS Analysis

SEM-EDS analysis was conducted to characterize the microstructural morphology, interfacial features, and local elemental composition of cement-stabilized macadam, thereby providing microscopic evidence for interpreting the changes in mechanical and shrinkage properties induced by DX-3. SEM observations were conducted using a field-emission scanning electron microscope (SU8010, Hitachi High-Tech Corporation, Tokyo, Japan) equipped with an energy-dispersive X-ray spectroscopy system (Oxford Instruments, Oxford, UK). Specimens with a cement dosage of 4.5% and DX-3 dosages of 0% and 1.5% were selected at curing ages of 7 d and 28 d.

After reaching the specified curing age, representative samples with dimensions of approximately 5 mm × 5 mm × 5 mm were collected from the specimens. The samples were dried, mounted on the specimen holders, and gold-coated prior to observation. SEM was used to examine the morphology and distribution characteristics of hydration products and the interfacial microstructure, while EDS was used to determine the elemental composition of representative regions. For qualitative EDS comparison, the analyzed regions were selected from the cementitious matrix using consistent selection criteria. Obvious coarse aggregate particles, large pores, and visually distinct foreign particles were avoided, and regions with comparable matrix morphology were preferentially selected. Because of the heterogeneous nature of cement-stabilized macadam, the EDS spectra were used only for qualitative comparison of local elemental characteristics. Particular attention was paid to changes in the relative distributions of Ca, Si, S, and other major elements associated with the observed microstructural characteristics.

## 3. Results and Discussion

### 3.1. Compressive Strength Performance

**Figure 5:**
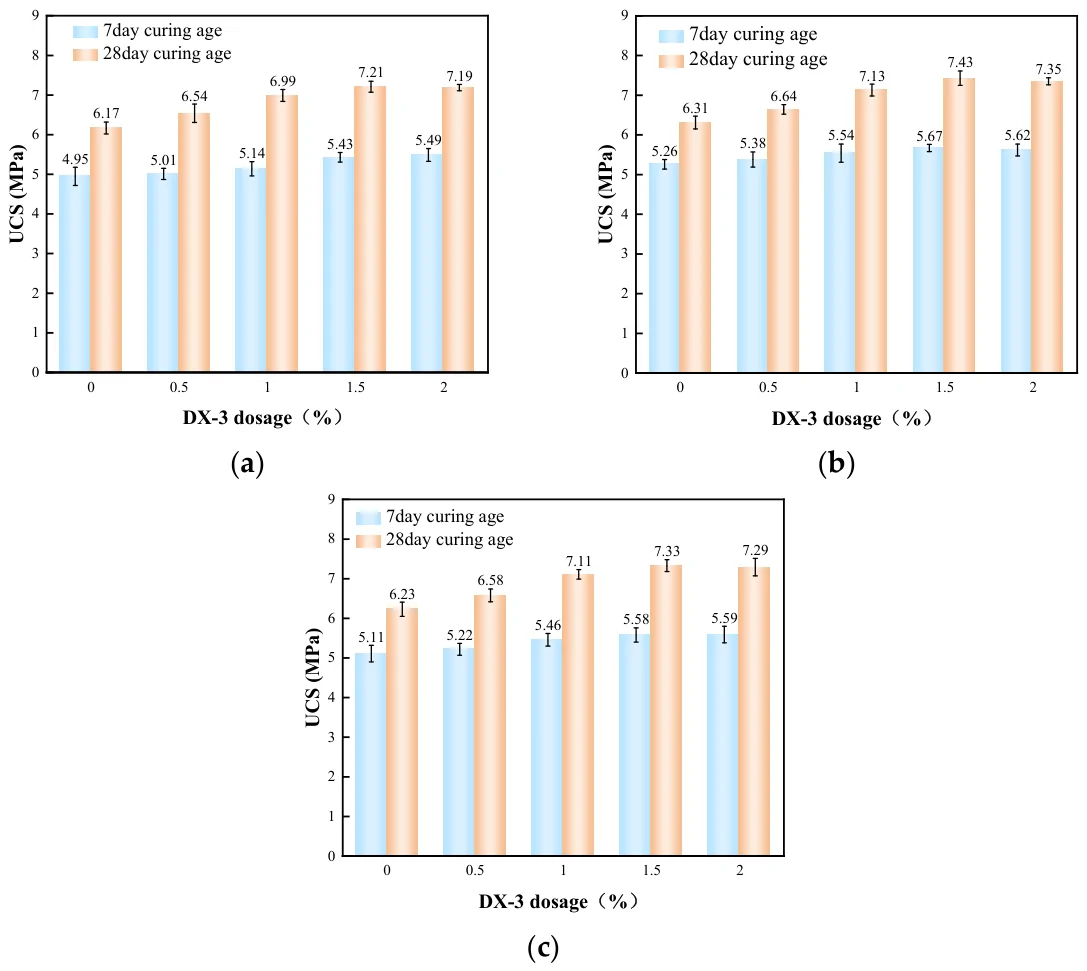
Compressive strength with different cement contents: (**a**) 4%; (**b**) 4.5%; (**c**) 5%. Error bars represent standard deviations of replicate measurements.

Figure 5 shows the UCS of cement-stabilized macadam with different cement and DX-3 dosages at curing ages of 7 d and 28 d. Overall, the UCS increased with curing age for all mixtures. At a fixed cement dosage, the strength generally increased with increasing DX-3 dosage up to 1.5%, followed by a tendency to stabilize or slightly decrease at 2.0%.

At 7 d, the differences among mixtures with different DX-3 dosages were relatively small. The highest UCS of 5.67 MPa was obtained at 4.5% cement and 1.5% DX-3. After 28 d of curing, the strength of all mixtures increased, and the effect of DX-3 became more evident. The mixture containing 4.5% cement and 1.5% DX-3 again exhibited the highest UCS, reaching 7.43 MPa. These results indicate that an appropriate amount of DX-3 does not impair the basic load-bearing capacity of cement-stabilized macadam and may provide a moderate strength benefit.

The increase in strength with curing age was mainly attributed to the continued development of the cementitious matrix. In contrast, the limited or slightly reduced strength at 2.0% DX-3 suggests that excessive modifier addition may not provide further mechanical benefit. Considering that 1.5% DX-3 consistently produced relatively high UCS values while 2.0% showed little additional improvement, 1.5% was selected as the representative dosage for the subsequent shrinkage and microscopic analyses.

### 3.2. Drying Shrinkage Performance

**Figure 6:**
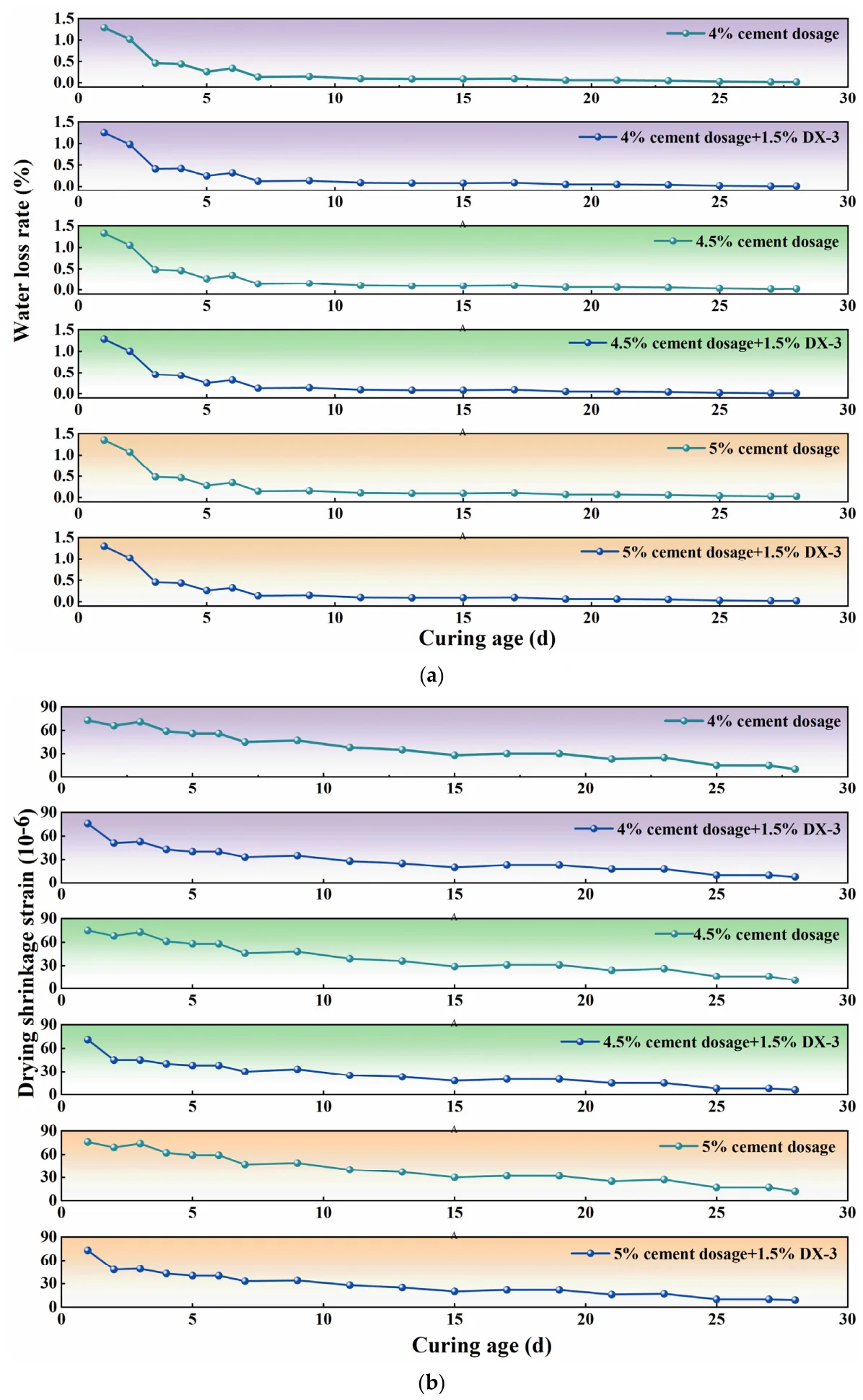

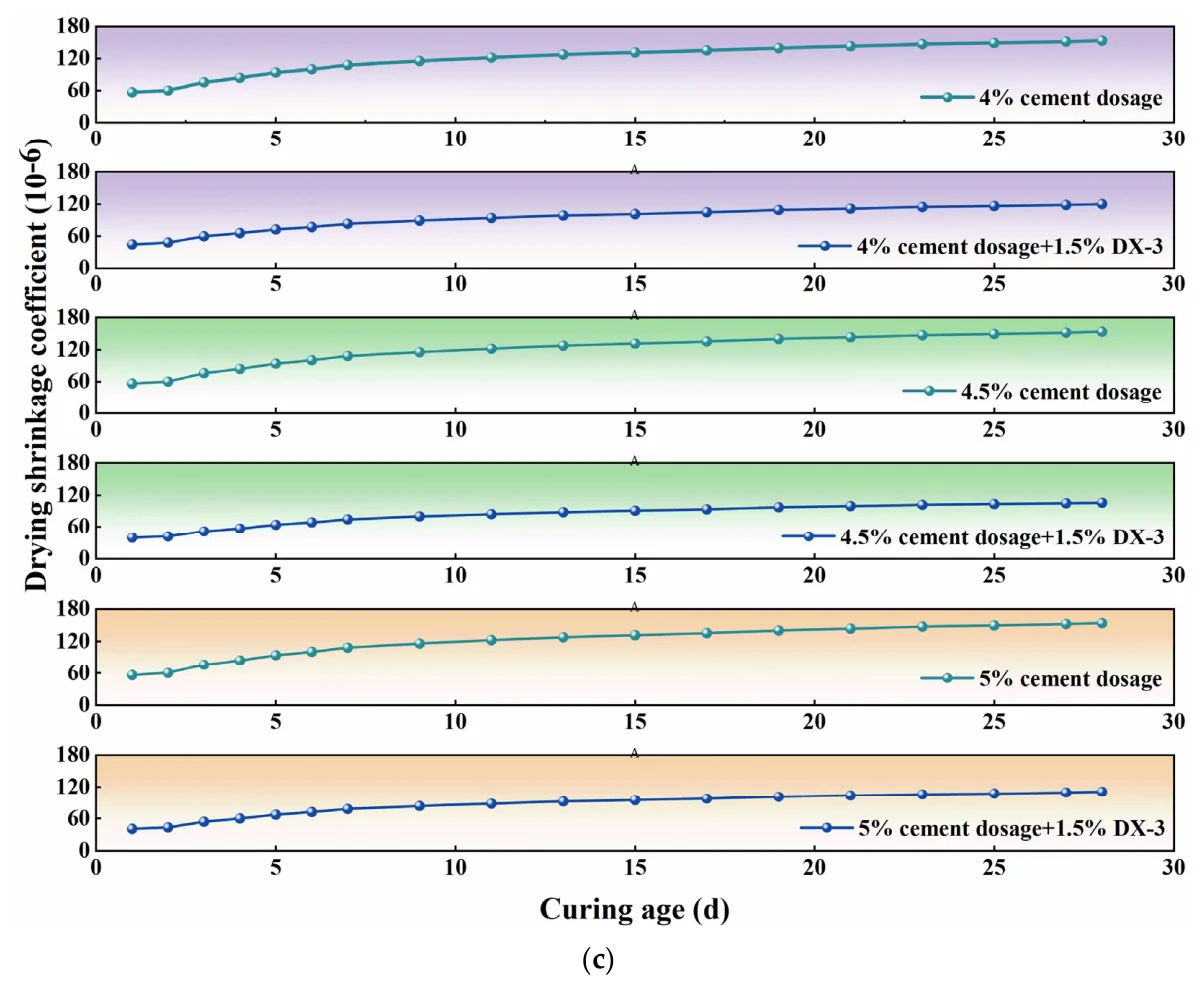
Drying shrinkage behavior of cement-stabilized macadam: (**a**) water-loss rate; (**b**) drying shrinkage strain; (**c**) drying shrinkage coefficient.

Figure 6 presents the evolution of the water-loss rate, drying shrinkage strain, and drying shrinkage coefficient with drying age. As shown in Figure 6a, moisture loss developed rapidly during the early drying stage and gradually slowed with increasing age. The incorporation of 1.5% DX-3 affected the water-loss process to some extent; however, the difference in moisture loss between the modified and control mixtures was relatively limited. In contrast, a more pronounced difference was observed in the drying shrinkage strain, as shown in Figure 6b. The shrinkage strain increased rapidly at the early stage and gradually tended to stabilize with increasing drying age. At the same cement dosage, the mixtures containing 1.5% DX-3 generally exhibited lower drying shrinkage strain than the corresponding control mixtures, indicating that DX-3 effectively reduced the volumetric deformation induced by moisture loss. The evolution of the drying shrinkage coefficient is shown in Figure 6c, and the corresponding total drying shrinkage coefficients are summarized in Table 8. After the incorporation of 1.5% DX-3, the total drying shrinkage coefficients decreased from 152.97 to 120.01 με/%, from 153.11 to 105.75 με/%, and from 153.75 to 110.66 με/% for cement dosages of 4.0%, 4.5%, and 5.0%, corresponding to reductions of 21.54%, 30.93%, and 28.03%, respectively. Among the investigated cement dosages, the greatest reduction was observed at 4.5% cement.

Notably, the reduction in drying shrinkage deformation was more pronounced than the change in moisture loss. This indicates that the improvement in drying shrinkage performance cannot be attributed solely to a reduction in water evaporation. Instead, the incorporation of DX-3 appears to reduce the shrinkage sensitivity of cement-stabilized macadam to moisture loss, resulting in a lower shrinkage response under comparable moisture-loss conditions.

### 3.3. Temperature Shrinkage Performance

**Figure 7:**
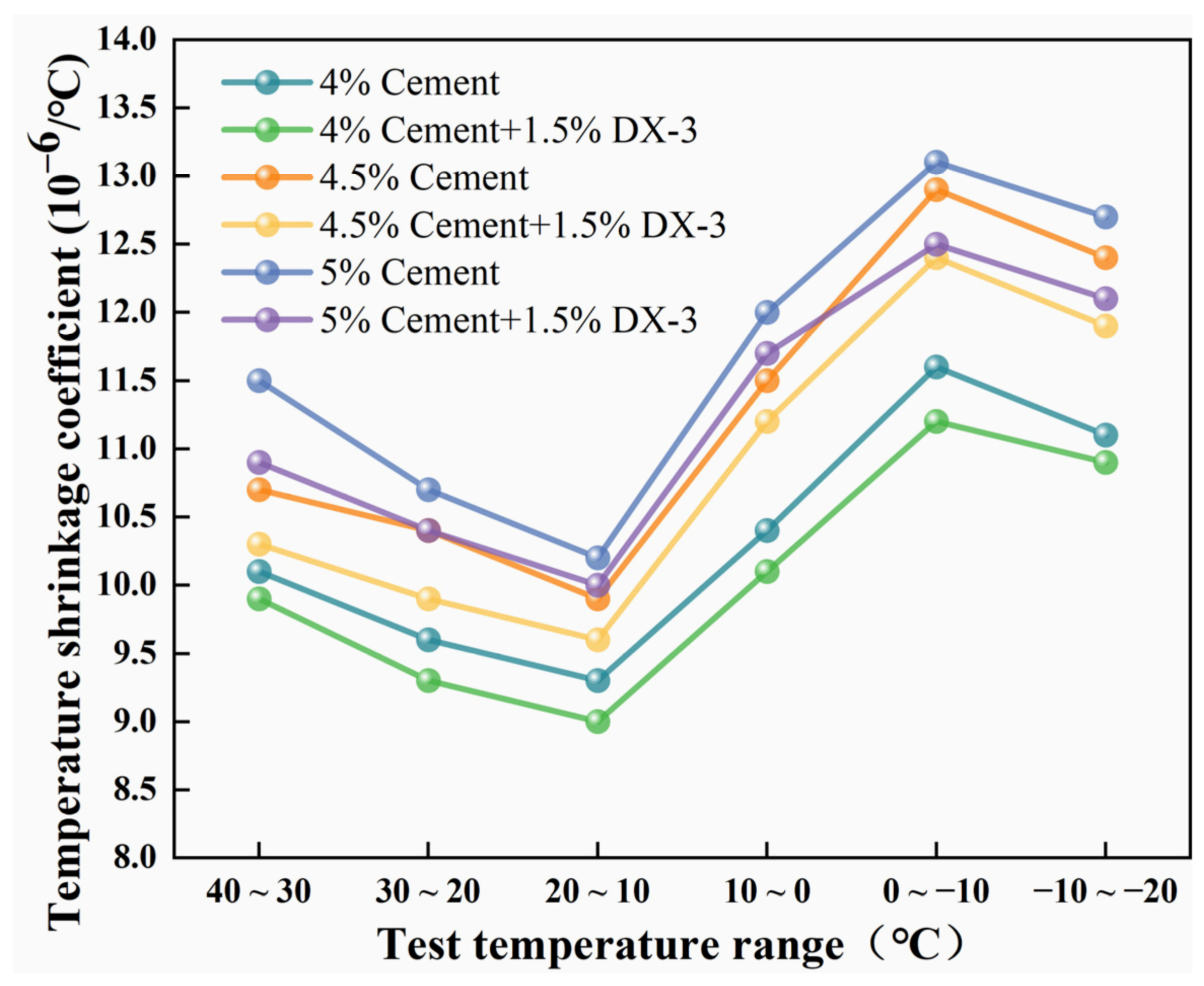
Temperature shrinkage test results.

Figure 7 presents the temperature shrinkage coefficients of the different mixtures over the investigated temperature intervals. All mixtures exhibited a similar temperature-dependent response. The temperature shrinkage coefficient initially decreased as the temperature decreased from 40 °C to approximately 10 °C, followed by a pronounced increase below 10 °C. The maximum values were observed in the temperature interval of 0 to −10 °C, after which the coefficients decreased slightly from −10 to −20 °C. This indicates that the temperature shrinkage behavior of cement-stabilized macadam is strongly temperature-dependent, with the region near the freezing point exhibiting the greatest shrinkage sensitivity. This behavior may be associated with changes in the state of pore water and the resulting internal deformation near the freezing point. The temperature shrinkage coefficient was also affected by cement dosage. As summarized in Table 9, for mixtures without DX-3, the average temperature shrinkage coefficient increased from 10.35 × 10^−6^ °C^−1^ at 4.0% cement to 11.30 × 10^−6^ °C^−1^ at 4.5% cement and 11.70 × 10^−6^ °C^−1^ at 5.0% cement. The corresponding peak values increased from 11.6 to 12.9 and 13.1 × 10^−6^ °C^−1^, respectively. These results indicate that increasing the cement dosage increased the sensitivity of the mixtures to temperature-induced volumetric deformation.

The incorporation of 1.5% DX-3 reduced the temperature shrinkage response at all three cement dosages. The average temperature shrinkage coefficients decreased by 2.71%, 3.72%, and 3.68% at cement dosages of 4.0%, 4.5%, and 5.0%, respectively. Meanwhile, the corresponding peak values decreased by 3.45%, 3.88%, and 4.58%. The reduction in both the average and peak coefficients indicates that DX-3 not only decreased the overall sensitivity of cement-stabilized macadam to temperature variations but also moderated the shrinkage response under the most unfavorable temperature interval. Together with the drying shrinkage results, these findings demonstrate that DX-3 improves the volumetric stability of cement-stabilized macadam under both moisture-loss and temperature-variation conditions while maintaining adequate compressive strength. The microstructural characteristics associated with these performance changes are further discussed in Section 3.4.

### 3.4. Microstructure Analysis

#### 3.4.1. Scanning Electron Microscopy (SEM) Analysis

**Figure 8:**
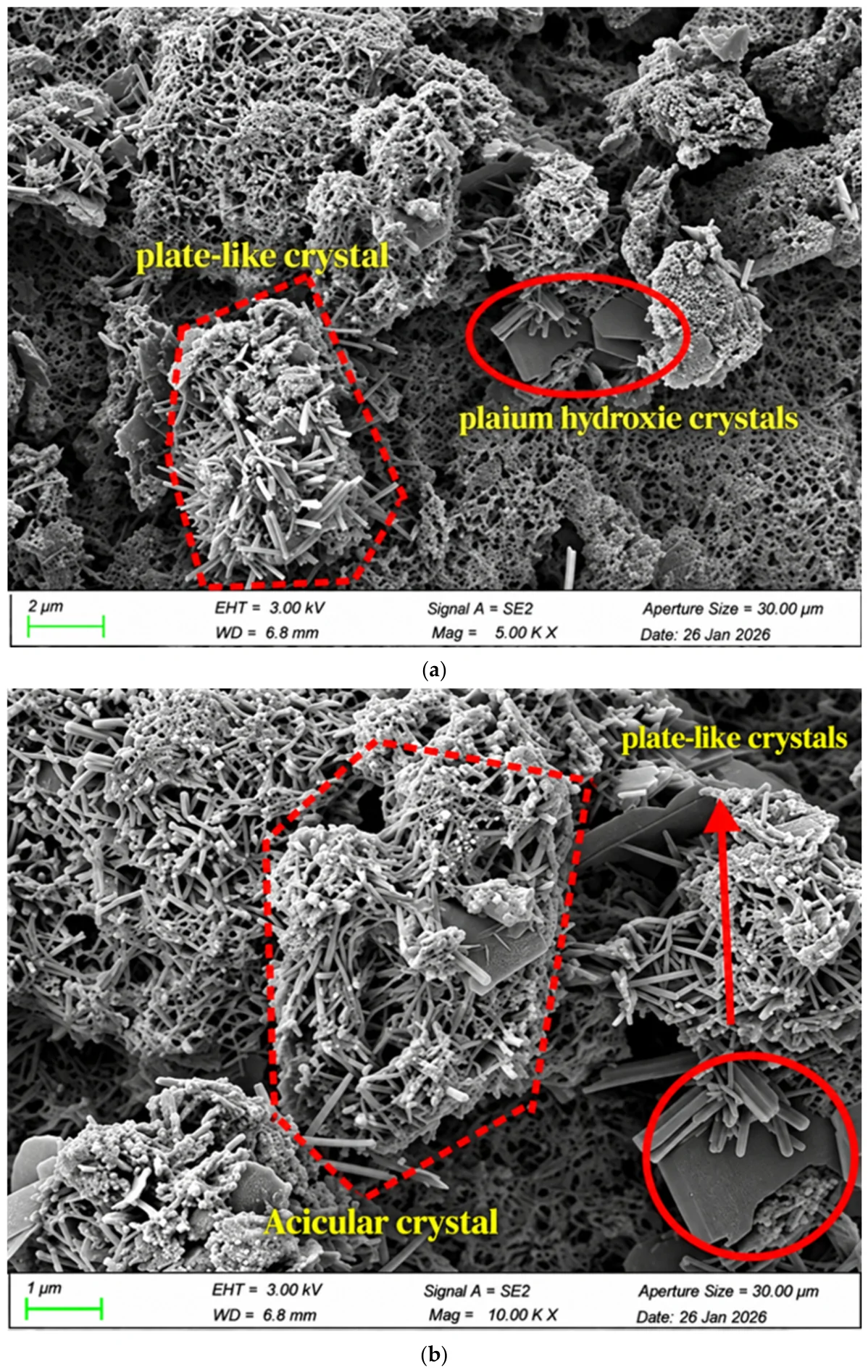

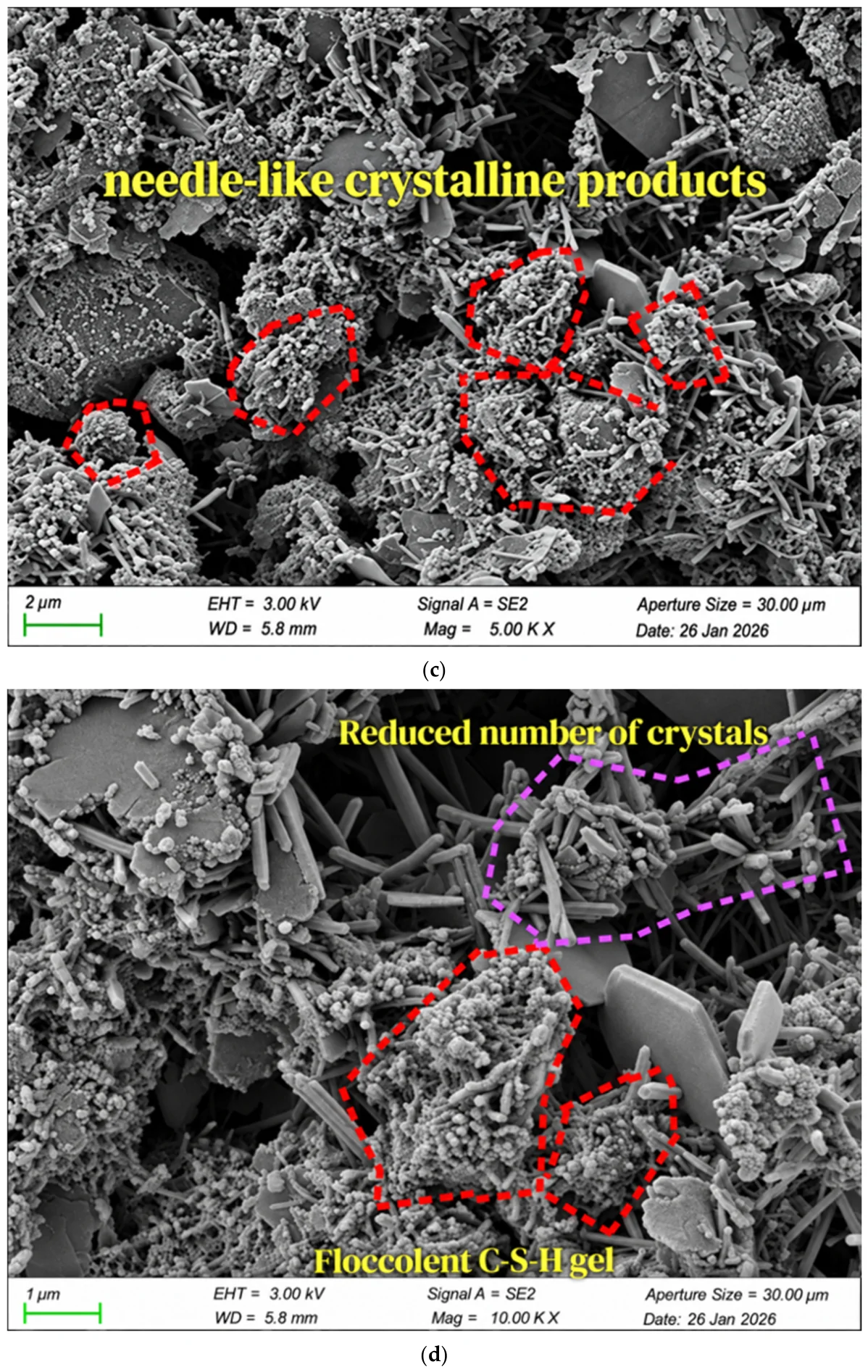

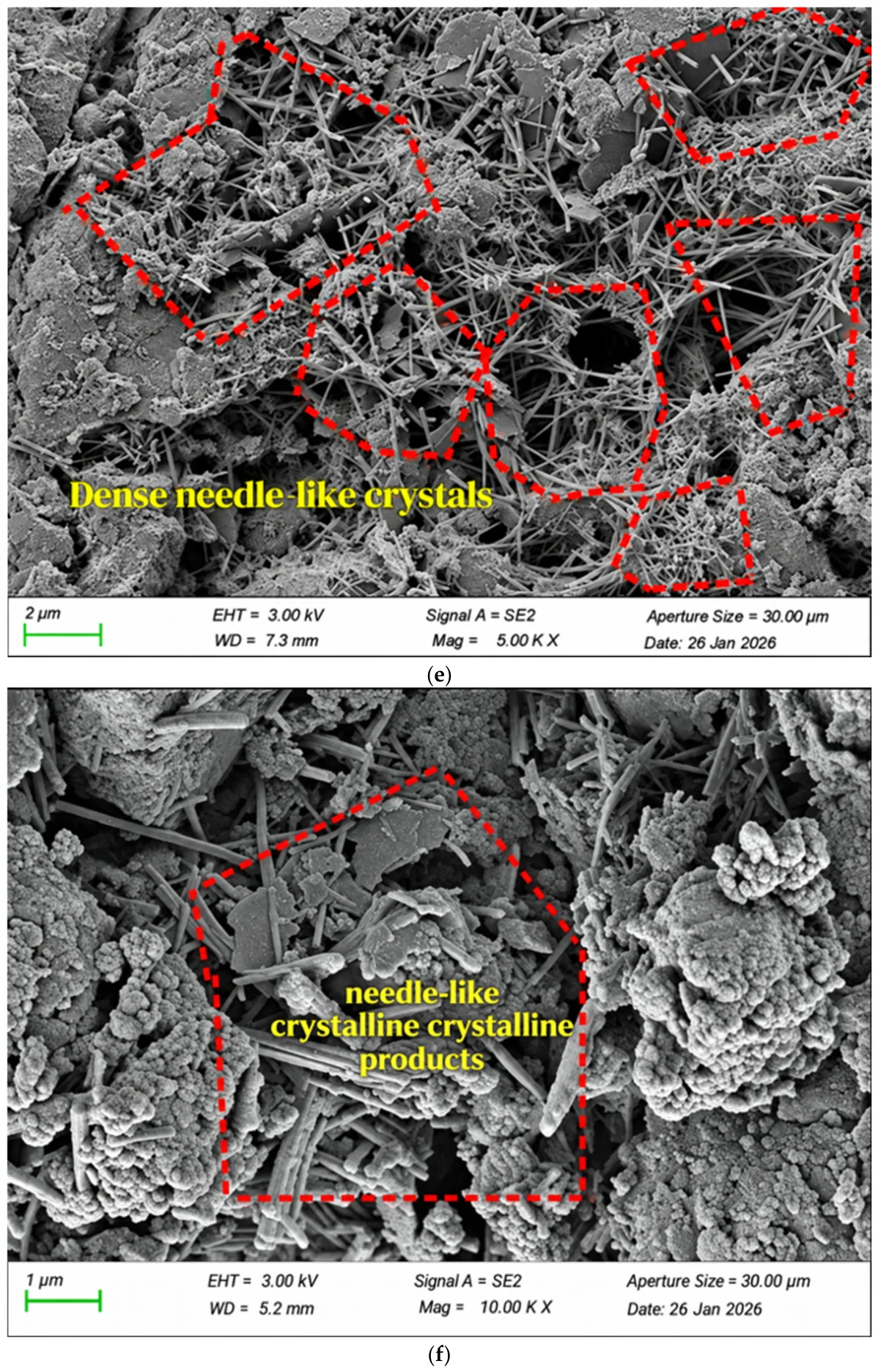

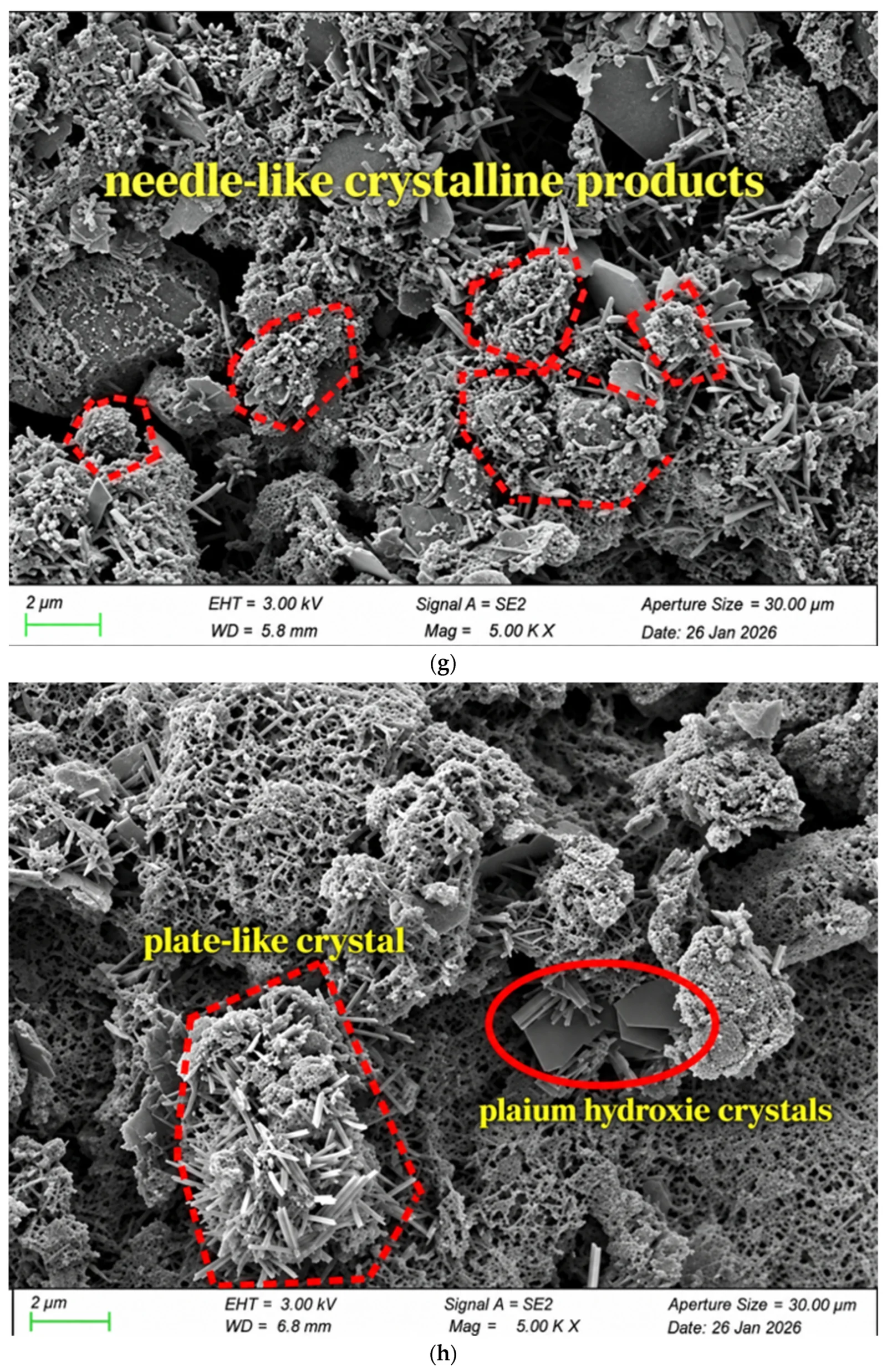
SEM micrographs of cement-stabilized macadam with different DX-3 dosages and curing ages: (**a**,**b**) 7 d, 0% DX-3; (**c**,**d**) 7 d, 1.5% DX-3; (**e**,**f**) 28 d, 0% DX-3; and (**g**,**h**) 28 d, 1.5% DX-3. The magnifications are 5000× and 10,000×, respectively (Dashed boxes/circles indicate representative microstructural regions discussed in the text, and arrows indicate the corresponding morphological features).

Figure 8 presents the SEM micrographs of cement-stabilized macadam with 0% and 1.5% DX-3 at curing ages of 7 d and 28 d. At 7 d, the specimen without DX-3 exhibited a relatively loose and heterogeneous microstructure, as shown in Figure 8a,b. Numerous needle-like and plate-like crystalline features were distributed within the cementitious matrix, accompanied by visible pores and discontinuities. In contrast, after the incorporation of 1.5% DX-3, more flocculent and gel-like features were observed, and these products appeared to be more closely connected, resulting in a relatively compact and continuous microstructure. (Figure 8c,d).

With increasing curing age, further development of the microstructure was observed. At 28 d, the specimen without DX-3 still contained abundant densely distributed needle-like crystals, with local pores and discontinuous regions remaining visible (Figure 8e,f). In comparison, the specimen containing 1.5% DX-3 exhibited more gel-like regions distributed among the crystalline phases. (Figure 8g,h). These regions partially filled interparticle spaces and contributed to a more continuous and compact microstructural morphology.

Comparison between the two curing ages indicates that the microstructure of both mixtures developed progressively from 7 d to 28 d, which is consistent with the continued evolution of the cementitious matrix during curing. The DX-3-modified specimens exhibited more gel-like regions and a more continuous matrix morphology than the corresponding control specimens. These observations suggest that DX-3 may influence the development of the cementitious matrix and reduce local deformation concentration. However, the SEM observations alone cannot identify specific hydration phases or quantitatively determine changes in hydration products. Therefore, the observed microstructural differences should be regarded as possible evidence associated with the improved mechanical and shrinkage performance rather than direct proof of a specific hydration mechanism.

#### 3.4.2. Energy-Dispersive X-Ray Spectroscopy (EDS) Analysis

EDS was adopted to conduct qualitative elemental analysis of specific regions observed by SEM. The chemical composition was determined to provide complementary elemental information for interpreting the observed microstructural features. A qualitative comparison of the characteristic EDS elemental signals is summarized in Table 10.

**Figure 9:**
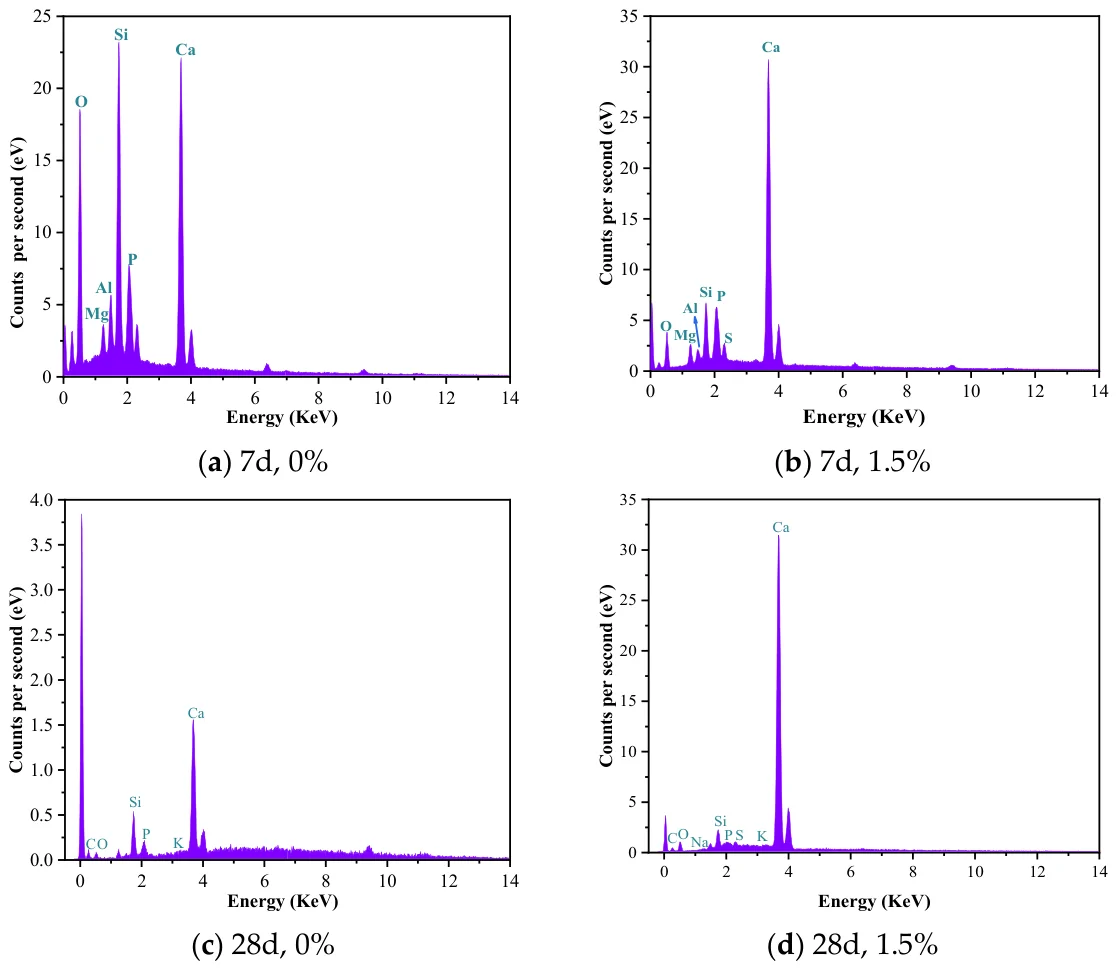
EDS spectra of cement-stabilized macadam with different DX-3 dosages and curing ages.

Figure 9 presents representative EDS spectra obtained from the control and DX-3-modified specimens after 7 and 28 d of curing. Differences in the relative intensities of Ca-, Si-, S-, and other elemental signals were observed among the analyzed regions. In particular, the examined DX-3-modified regions exhibited a relatively more pronounced Si-related signal and a weaker S-related signal than the corresponding control regions. However, because EDS provides local elemental information and is sensitive to the selected analysis region, these differences cannot be uniquely attributed to changes in specific hydration products. Contributions from locally distributed aggregate/mineral phases and DX-3 constituents cannot be completely excluded. Therefore, the EDS results are used only as qualitative evidence of differences in local elemental characteristics between the examined regions and not as direct evidence of changes in the amount or type of individual hydration phases.

Overall, the qualitative EDS results are consistent with the SEM observations. The incorporation of DX-3 was associated with changes in the local elemental characteristics and a greater prevalence of gel-like microstructural features. It should be emphasized that the SEM-EDS results provide primarily morphological and local elemental information. Although the DX-3-modified specimens exhibited a more continuous matrix with a greater prevalence of gel-like features, and EDS confirmed the presence of Ca and Si in representative regions, these observations alone are insufficient to conclusively identify specific hydration phases or quantify changes in hydration reactions. Therefore, the present SEM-EDS results should be interpreted as evidence of microstructural differences associated with DX-3 incorporation rather than direct proof that DX-3 promotes the formation of a particular hydration product. Complementary phase and thermal characterization, such as XRD and TG/DTG, will be required in future work to verify the proposed hydration-related mechanisms.

## 4. Conclusions

A self-developed DX-3 composite modifier was investigated to evaluate its effects on the mechanical strength, shrinkage behavior, and microstructural characteristics of cement-stabilized macadam. Based on the experimental results, the following conclusions can be drawn:

(1) Within the investigated dosage range, DX-3 did not adversely affect the basic compressive strength of cement-stabilized macadam. The UCS generally increased as the DX-3 dosage increased to 1.5% and then tended to stabilize or slightly decrease at 2.0%. At 4.5% cement and 1.5% DX-3, the 7 d and 28 d UCS reached 5.67 and 7.43 MPa, respectively. Considering its consistently favorable strength response, 1.5% DX-3 was selected as the representative dosage for subsequent shrinkage and microscopic analyses.
(2) DX-3 effectively reduced drying-shrinkage-related deformation. At cement dosages of 4.0%, 4.5%, and 5.0%, the incorporation of 1.5% DX-3 reduced the total drying shrinkage coefficient by 21.54%, 30.93%, and 28.03%, respectively. The reduction in shrinkage deformation was more pronounced than the change in moisture loss, indicating that DX-3 mainly reduced the shrinkage sensitivity of the material to moisture loss rather than simply suppressing water evaporation.
(3) The temperature shrinkage behavior exhibited an evident temperature dependence, with the highest shrinkage sensitivity occurring in the 0 to −10 °C interval. The incorporation of 1.5% DX-3 reduced the average temperature shrinkage coefficient by 2.71–3.72% and the peak coefficient by 3.45–4.58% at different cement dosages. These results indicate that DX-3 can reduce both the overall temperature sensitivity and the maximum shrinkage response under unfavorable temperature conditions.
(4) SEM observations showed that the DX-3-modified specimens exhibited more extensive gel-like morphological features and a relatively continuous and compact cementitious matrix in the examined regions. Qualitative EDS analysis revealed differences in local elemental signals between the modified and control specimens; however, these differences cannot be used to identify or quantify specific hydration phases. The SEM-EDS observations therefore provide complementary evidence of microstructural differences associated with DX-3 incorporation rather than direct proof of a particular hydration mechanism.

This study has several limitations that should be considered when interpreting the results. First, the experimental program was conducted under controlled laboratory conditions using a specific aggregate gradation, cement type, and range of DX-3 dosages; therefore, the applicability of the observed effects to other material systems and field conditions requires further verification. Second, the present study focused primarily on compressive strength and shrinkage behavior, whereas long-term durability and cracking performance under coupled environmental and traffic loading were not evaluated. Third, the microstructural interpretation was mainly based on SEM observations and qualitative EDS analysis and therefore cannot conclusively identify or quantify individual hydration products. Future studies should evaluate the long-term durability and field performance of DX-3-modified cement-stabilized macadam, optimize the modifier formulation for different material systems, and employ complementary techniques such as XRD, TG/DTG, and quantitative microstructural analysis to further clarify the underlying modification mechanisms. Moreover, a systematic comparison with commercial modifiers regarding cost, long-term durability, and field applicability should be addressed in future research.

## Figures and Tables

**Table 1:** Physical properties of cement.

Test Items		Specified Value	Results
Standard consistency water demand (%)		/	27.8
Soundness (boiling method)		After boiling C-A ≤ 5.0 mm	0.5
Initial setting time (min)		>180 min	220
Final setting time (min)		>360 and <600	368
Compressive strength (MPa)	3 d	≥17	28.9
	28 d	≥42.5	50.0
Flexural strength (MPa)	3 d	≥3.5	4.9
	28 d	≥6.5	7.6

Note: The data were obtained from the supplier.

**Table 2:** Aggregate test results.

Particle Size	Test Items	Specified Value	Results
19–31.5 mm	Needle and flaky particle content (%)	≤22	3.6
	0.075 mm passing content (%)	≤2	0.2
	Crushing value (%)	≤26	23.7
	Apparent density (g/cm^3^)	≥2.5	2.772
	Water absorption (%)	≤2.0	0.42
9.5–19 mm	Needle and flaky particle content (%)	≤22	12.3
	0.075 mm passing content (%)	≤2	0.3
	Crushing value (%)	≤26	21.4
	Apparent density (g/cm^3^)	≥2.5	2.846
	Water absorption (%)	≤2.0	0.48
4.75–9.5 mm	Needle and flaky particle content (%)	≤22	4.2
	0.075 mm passing content (%)	≤2	0.1
	Apparent density (g/cm^3^)	≥2.5	2.821
	Water absorption (%)	≤2.0	0.53
2.36–4.75 mm	0.075 mm passing content (%)	≤15	0.2
	Apparent density (g/cm^3^)	≥2.5	2.765
	Water absorption (%)	≤2.0	0.60
0–2.36 mm	0.075 mm passing content (%)	≤15	11.3
	Apparent density (g/cm^3^)	≥2.5	2.704
	Water absorption (%)	≤2.0	0.67

Note: The aggregate properties were obtained from the supplier.

**Table 3:** Composition of the DX-3 composite modifier.

Component	Content Range (wt.%)	Function
Deionized water	60–70	Reaction medium and dispersion phase
Methallyl polyoxyethylene ether	8–15	Polyether functional monomer
Methacrylic acid	3–8	Carboxyl functional monomer
Cellulose	2–5	Rheological modification and water retention
Polymer nanomaterials	3–8	Microstructure modification component
Propylene glycol	3–6	Stabilizing and dispersing agent
Other composite additives	1–3	Auxiliary modification components

**Table 4:** Basic physicochemical properties of the DX-3 composite modifier.

Test Item	Technical Requirement	Result
pH value	≥4.5	6.2
Chloride content (mg/L)	≤350	85
Sulfate content (mg/L)	≤270	72
Total sulfur content (%)	≤1.5	0.85
Density (g/mL)	≥1.05	1.12
Solid content (%)	≥25	32.6

**Table 5:** Optimum moisture content, maximum dry density, and compaction characteristics of the mixtures.

Cement Dosage (%)	DX-3 (%)	Optimum Moisture Content(%)	Maximum Dry Density(g/cm^3^)	Dry Density (g/cm^3^)	Compaction Degree (%)
4.0	0	4.8	2.443	2.394	98.0
	0.5			2.399	98.2
	1			2.392	97.9
	1.5			2.397	98.1
	2			2.394	98.0
4.5	0	4.9	2.451	2.404	98.1
	0.5			2.402	98.0
	1			2.407	98.2
	1.5			2.400	97.9
	2			2.404	98.1
5.0	0	4.8	2.482	2.432	98.0
	0.5			2.435	98.1
	1			2.430	97.9
	1.5			2.437	98.2
	2			2.432	98.0

**Table 6:** Analysis of variance of DX-3.

Source	SS	df	MS	F	*p*-Value
Between DX-3 dosages	0.00001227	4	0.00000307	0.0075	0.9999
Within groups	0.00407533	10	0.00040753	/	
Total	0.00408760	14	/		

**Table 7:** The final quantities of raw materials for all mixtures.

Cement Dosage (%)	DX-3 Dosage (% of Cement)	Cement (kg)	DX-3 (kg)	Water (kg)
4.0	0	40	0	49.92
	0.5		0.2	
	1.0		0.4	
	1.5		0.6	
	2.0		0.8	
4.5	0	45	0	52.21
	0.5		0.2	
	1.0		0.4	
	1.5		0.6	
	2.0		0.8	
5.0	0	50	0	50.40
	0.5		0.2	
	1.0		0.4	
	1.5		0.6	
	2.0		0.8	

Note: Mass based on 1000 kg of dry aggregate.

**Table 8:** Total drying shrinkage coefficient of different mixtures (με/%).

Cement Dosage (%)	Total Drying Shrinkage Coefficient		Reduction (%)
	0% DX-3 Dosage	1.5% DX-3 Dosage	
4	152.97	120.01	21.54
4.5	153.11	105.75	30.93
5	153.75	110.66	28.03

**Table 9:** Average and peak temperature shrinkage coefficients of different mixtures.

Cement Dosage (%)	DX-3 Dosage (%)	Average Temperature Shrinkage Coefficient (×10^−6^ °C^−1^)	Reduction (%)	Peak Temperature Shrinkage Coefficient (×10^−6^ °C^−1^)	Reduction (%)
4.0	0	10.35	—	11.6	—
4.0	1.5	10.07	2.71	11.2	3.45
4.5	0	11.30	—	12.9	—
4.5	1.5	10.88	3.72	12.4	3.88
5.0	0	11.70	—	13.1	—
5.0	1.5	11.27	3.68	12.5	4.58

**Table 10:** Qualitative comparison of characteristic EDS elemental signals for cement-stabilized macadam with different DX-3 dosages and curing ages.

Curing Age	DX-3 Dosage	Ca Signal	Si Signal	S Signal	Qualitative Observation
7 d	0%	Strong	Relatively weak	Evident	Local elemental characteristics of the control matrix
7 d	1.5%	Strong	Enhanced	Weakened	Different local Si- and S-related signals
28 d	0%	Strong	Moderate	Evident	Local elemental characteristics of the control matrix
28 d	1.5%	Strong	Further enhanced	Weak	Different local Si- and S-related signals

## Data Availability

The original contributions pre-sented in this study are included in the article. Further inquiries can be directed to the cor-responding authors.
